# A Mobile App Leveraging Citizenship Engagement to Perform Anonymized Longitudinal Studies in the Context of COVID-19 Adverse Drug Reaction Monitoring: Development and Usability Study

**DOI:** 10.2196/38701

**Published:** 2022-11-04

**Authors:** Marzia Di Filippo, Alessandro Avellone, Michael Belingheri, Maria Emilia Paladino, Michele Augusto Riva, Antonella Zambon, Dario Pescini

**Affiliations:** 1 Department of Statistics and Quantitative Methods University of Milano-Bicocca Milan Italy; 2 School of Medicine and Surgery University of Milano-Bicocca Monza Italy

**Keywords:** ADR reporting, adverse drug reaction–reporting systems, mobile apps, longitudinal studies, COVID-19 vaccination campaign, COVID-19, vaccine, apps, adverse drug reaction, pharmacovigilance, anonymity

## Abstract

**Background:**

Over the past few years, studies have increasingly focused on the development of mobile apps as complementary tools to existing traditional pharmacovigilance surveillance systems for improving and facilitating adverse drug reaction (ADR) reporting.

**Objective:**

In this research, we evaluated the potentiality of a new mobile app (vaxEffect@UniMiB) to perform longitudinal studies, while preserving the anonymity of the respondents. We applied the app to monitor the ADRs during the COVID-19 vaccination campaign in a sample of the Italian population.

**Methods:**

We administered vaxEffect@UniMiB to a convenience sample of academic subjects vaccinated at the Milano-Bicocca University hub for COVID-19 during the Italian national vaccination campaign. vaxEffect@UniMiB was developed for both Android and iOS devices. The mobile app asks users to send their medical history and, upon every vaccine administration, their vaccination data and the ADRs that occurred within 7 days postvaccination, making it possible to follow the ADR dynamics for each respondent. The app sends data over the web to an application server. The server, along with receiving all user data, saves the data in a SQL database server and reminds patients to submit vaccine and ADR data by push notifications sent to the mobile app through Firebase Cloud Messaging (FCM). On initial startup of the app, a unique user identifier (UUID) was generated for each respondent, so its anonymity was completely ensured, while enabling longitudinal studies.

**Results:**

A total of 3712 people were vaccinated during the first vaccination wave. A total of 2733 (73.6%) respondents between the ages of 19 and 80 years, coming from the University of Milano-Bicocca (UniMiB) and the Politecnico of Milan (PoliMi), participated in the survey. Overall, we collected information about vaccination and ADRs to the first vaccine dose for 2226 subjects (60.0% of the first dose vaccinated), to the second dose for 1610 subjects (43.4% of the second dose vaccinated), and, in a nonsponsored fashion, to the third dose for 169 individuals (4.6%).

**Conclusions:**

vaxEffect@UniMiB was revealed to be the first attempt in performing longitudinal studies to monitor the same subject over time in terms of the reported ADRs after each vaccine administration, while guaranteeing complete anonymity of the subject. A series of aspects contributed to the positive involvement from people in using this app to report their ADRs to vaccination: ease of use, availability from multiple platforms, anonymity of all survey participants and protection of the submitted data, and the health care workers’ support.

## Introduction

Pharmacovigilance is defined by the World Health Organization (WHO) as “the science and activities relating to the detection, assessment, understanding, and prevention of adverse effects or any other drug-related problem” [1]. Monitoring the safety of medicines is fundamental because many previously undetected adverse drug reactions (ADRs) during clinical trials may occur when patients are exposed to drugs. ADRs refer to any undesired effect emerged during treatment with a given pharmaceutical product [2].

We performed a literature analysis to review the currently adopted methods for ADR reporting. In particular, we searched PubMed for papers published in the past 10 years using the following query: “adverse drug reactions” OR “adverse drug reaction reporting” OR “adverse drug reaction reporting systems” (query performed on June 22, 2022). The considered papers included clinical trials, meta-analyses, randomized controlled trials, and reviews. This search identified 131 studies. By excluding papers that, after a full text or abstract screening, seemed too descriptive and generic or focused on just presenting the incidence or prevalence of ADRs, the literature survey performed was limited to 28 eligible papers. We found 9 other papers that met the considered criteria. We grouped the studies based on the system used to conduct ADR surveillance.

Of the total 39 papers found, 7 (17.9%) [3-9] are based on traditional safety surveillance. This approach relied on data collected through passive reports of ADRs from consumers and health providers, medical literature, and observational databases [10]. This way, any active measure is taken to search for ADRs, except for voluntary and spontaneous reports by health care providers or vaccinees [11,12]. Although passive reporting is still used, thanks to the possibility of identifying rare and unexpected ADRs, its ease of implementation, and relatively low cost, it can be limited by underreporting, lack of information, and difficulty in determining ADR rates [12,13]. Moreover, traditional surveillance of ADRs after drug exposure is slow and patchy. Indeed, once reported by patients or health care professionals, ADRs are first assessed by drug experts and pharmaceutical companies, and then the result of the assessment is passed on to government agencies. This path may entail underreporting issues, with considerable data loss.

Substantial delays may occur between occurrence and discovery of ADRs, because reports received by government agencies are often released with a delay of months or even years due to the period required for proper assessment of ADR data. Further limitations of traditional ADR surveillance relate to the fact that ADR reporting is voluntary, and several studies have shown that as many as 90% of serious ADRs end up being unreported [2,14]. Moreover, several studies have reported low awareness of the national public ADR reporting systems within the general population, with a portion of the public not even being aware of them. This adds up to a problem of declining reporting by physicians that has been linked to the belief that it is only necessary to report serious or unexpected ADRs or that a single observed ADR could not contribute to medical knowledge or that only ADRs derived from a certain causal relationship with the use of a particular drug should be reported [10,15].

In response to these limitations, ADR surveillance is also conducted by applying a participant-centered active approach that, contrary to passive reporting, involves an intentional searching for ADRs via continuous and organized contact with health care providers or other relevant reporting sources, such as hospitals, laboratories, and patients [11,12].

At the same time, active surveillance requires more resources for a positive outcome. Of the 39 papers, in 16 (41%), active systems falling under this category used digital strategies to facilitate and encourage ADR surveillance, which means the adoption of electronic technology for capturing and processing data.

Common participant-centered active surveillance methods are conducted using health diary reporting cards [11], SMS text messages (eg, SmartVax [16], VaxTracker [17], stimulated telephone-assisted rapid safety surveillance [STARSS] [18], FASTMum [19], FAST-Health [20]), and email (eg, VaxTracker [17], Lareb Intensive Monitoring [21], and integrated vaccine surveillance system [IVSS] [22]) to interact with individuals and prompt them to report their ADRs by sending a web link to complete an online questionnaire, interviews collected over the phone (eg, FASTMum [19], IVSS [22], and a project developed in Brazil [13,23,24]), during medical visits [25], or mailing questionnaires [26-29].

Thanks to the spread of internet use, our literature survey suggested increasing usage of web-based platforms to promote ADR reporting. A large proportion of the papers found (16/39, 41%) adopted interactive web-based ADR-reporting tools [30-33] also accessible from mobile devices [34] that linked to the receipt of spontaneous ADR reports [30], to the use of electronic health records to directly report ADRs [35-37], and to spontaneous reports sent through multiple forms of notification (eg, via an ADR portal, telephone, and email [38]) and sent on the online Yellow Card scheme [39-42]. The same explored ways are also used individually or in combination to report ADRs through a paper-based form sent by email or fax, an electronic form sent on a website, or telephone interviews [43,44]. This category of web-based platforms to report ADRs also includes the CANVAS program [45], which relies on reports received through a web-based survey, with an additional telephone follow-up when severe ADRs need to be reported.

The results of the European Union's Innovative Medicines Initiative Web–Recognising Adverse Drug Reactions (WEB-RADR) project, reported in a recent paper [46], suggest that the patient’s increased interest in improving knowledge about drug safety, combined with recent technological advances in information communication, make it possible to improve ADR reporting and patient safety communication using mobile apps.

Over the past few years, studies have increasingly focused on the development and usage of mobile apps as additional tools to pharmacovigilance for improving and facilitating reporting of ADRs, as reported by Defer et al [47], Ahn et al [48], Prakash et al [49], Montastruc et al [50], and de Vries et al [51]. Indeed, in contrast to classical surveillance systems, mobile devices offer a platform for improving the accessibility of data and increasing the speed at which they are transmitted between institutions. These tools lead to real-time systems of pharmacovigilance with the potential of enabling a near-instantaneous transmission of patient safety information through user-friendly and interactive graphics within the app [10]. The extensive usage of smartphones and other mobile devices with internet access leads to the expectation of a greater involvement from the public in using mobile health apps to report suspected ADRs, complementing traditional surveillance systems.

In view of these findings, we searched for available mobile apps with the ability to allow ADR reporting for medical devices, drugs, or vaccines, without searching for apps that are specific for a particular case of study.

Among the tools we found, the MedWatcher app [52] is a web-based and mobile app available to the US public, developed in partnership with the Food and Drug Administration (FDA) Center for Devices and Radiologic Health (CDRH) and available on Apple App Store and Google Play Store for iOS and Android devices, respectively. Through MedWatcher, reports are processed in a secure cloud computing environment, manually reviewed, and then transmitted electronically to the Manufacturer and User Facility Device Experience (MAUDE) database. VigiBIP [50], designed by the pharmacovigilance network in France and available for Android and iOS devices, has the notable functionality to allow reporting of not only data but also photographs and images [50,53]. Similarly, the ADR PvPi app [49], which was developed by the National Coordination Centre-Pharmacovigilance Programme of India, facilitates ADR reporting, enabling document and image attachment by health care professionals as well as consumers. However, it was available just on Android devices from the Google Play Store.

Another available tool is presented by Ahn et al [48], where the system design is accurately presented as consisting of a mobile app, a cloud server, and a dashboard. Patients’ data on symptoms and vaccination are captured using a mobile device or a web interface and then sent to the cloud server. Medical personnel then use the web dashboard to receive and analyze the submitted data. This mobile app was developed for both Android and iOS devices.

My eReport France [47] is a free mobile app available for both Android and iOS devices. When individuals use this app, they receive a participant card containing quick response (QR) codes to download the app and submit their ADR report.

The WEB-RADR project developed 3 mobile apps suitable for patients, caregivers, and health care professionals not only to report their ADRs but also to receive up-to-date information and news alerts on selected medicines, check the number of received reports for a particular drug, and view previous ADR reports submitted through the app. These 3 apps, which are free to use for everyone on iOS and Android devices, are the free Yellow Card smartphone app [42] developed in 2015, the Halmed app [54] launched in 2016 to submit ADRs to drugs directly to the Agency for Medicinal Products and Medical Devices of Croatia, and the Lareb app [55] developed in 2016 by the Netherlands Pharmacovigilance Centre Lareb but no longer maintained or available for download.

In the context of the WEB-RADR project, the international WEB-RADR app has been launched under the name Med Safety [56]. Similar to the other WEB-RADR apps, Med Safety is a smartphone app freely available on both iOS and Android devices for reporting ADRs to national competent authorities, keeping track of previously reported information and receiving news about drugs of interest. With the ability to function without an internet connection, individuals can partially create reports and save them for completion later or create and save reports without sending them immediately but subsequently once connectivity is re-established.

In the following cases, no specific details about the app design and features, other than the chosen platform and the declared ability to allow ADR reporting, are available: the CANVAS app [45] and the ADR online app [57], which are available only for iOS devices; the ADR Reporter app [58] and the TMDA Adverse Reactions Reporting Tool app [59], which are available only for Android devices; and the Easypharm app [60] and the UAE RADR app [61], which are available for both Android and iOS devices.

Although the development of mobile apps to report ADRs is continuing to progress, providing valuable knowledge about drug safety, one feature that is not present in any of the reviewed apps in our literature analysis is the ability to perform longitudinal analysis in an anonymous way, a key point to foster citizenship engagement.

Although having valuable features, all the currently available mobile apps lack the ability to collect individual subjects’ ADR time courses anonymously. Monitoring the same subjects over time, while guaranteeing complete privacy and anonymity of the submitted information, can be a valuable addition to pharmacovigilance because it can be used to gather longitudinal safety data of a drug. Consequently, the temporal investigation of the same patient can help detect, within a specific clinical context, any temporal patterns or ADR combinations.

On these grounds, we developed, for Android and iOS devices, a new, free app called vaxEffect@UniMiB, tested its capability to enable longitudinal studies by analyzing per subject time series, and evaluated its potential in fostering spontaneous citizenship participation in an ADR data collection campaign. In this work, we specifically presented app features by evaluating the reporting rate of ADRs in a sample of Italian academic subjects vaccinated for COVID-19.

## Methods

### Study Design

During the COVID-19 health emergency, the University of Milano-Bicocca (UniMiB) participated in a vaccination campaign deploying a hub. The hub administered the first dose between March 5 and 29, 2021, and the second dose between May 31 and June 11, 2021. A third dose was administered at the national level, but the authorities decided to not involve the hub. The campaign involved professors, researchers, adjunct professors, postdocs, PhD students, and the technical administrative staff of UniMiB or of the Politecnico of Milan (PoliMi). Due to the deputed authority emergency plan, the UniMiB hub invited all UniMiB employees and only a third of PoliMi ones, the others being distributed to different hubs.

### Ethical Considerations

Ethical approval was obtained from the Ethical Committee of UniMiB (protocol no. 0041302/21).

### Data Collection

After receiving ethical approval, vaxEffect@UniMiB was made available on March 24, 2021, from Google Play Store and on March 29, 2021, from Apple App Store. It was advertised to subjects invited to the vaccination campaign at the UniMiB hub.

We started to collect reports sent to vaxEffect@UniMiB, starting from January 1, 2021. Given the health emergency, it was not possible to plan the different phases of the study. To highlight the uncertainties of the situation, we report that the UniMiB hub was not aware at the end of the first dose administration whether the second dose would have been administered by the hub itself. When the third dose was administered at the national level, the hub was not involved, and we decided to test the flexibility of the app by keeping it active to allow respondents to use it on a spontaneous basis. The app was deactivated on February 9, 2022.

At each vaccination wave, the physicians involved in the vaccination campaign explained to attendees the importance of reporting ADRs and delivered a brochure explaining the aim of the research, as well as the guarantee of anonymization of the digital tool. Moreover, corresponding to the beginning of the first 2 vaccine doses’ administration campaign, an informative mail was sent to UniMiB employees.

### System Overview

**Figure 1:**
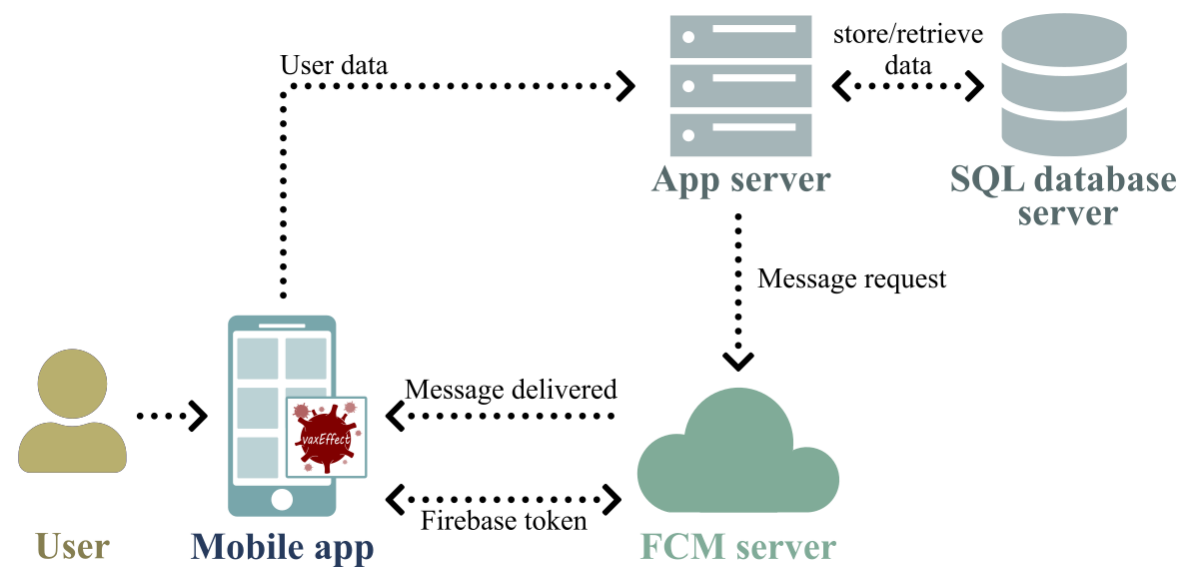
Our system. FCM: Firebase Cloud Messaging.

Our system consists of a mobile app, a web server, and a Firebase Cloud Messaging (FCM) server (Google Inc), as shown in Figure 1. At initial startup, the mobile app inquiries about the user’s health condition, focusing only on aspects relevant to the vaccination campaign, and sends the answers to the application server. Upon every vaccine administration, vaccine data and ADRs occurring within 7 days postvaccination are sent by the patient using the mobile app.

The application server is responsible for receiving all the user data, to store them in a SQL database server, and to remind the patients to submit vaccine and ADR data. The reminders are sent to the patients via push notifications sent to the mobile app by the application server through FCM.

**Figure 2:**
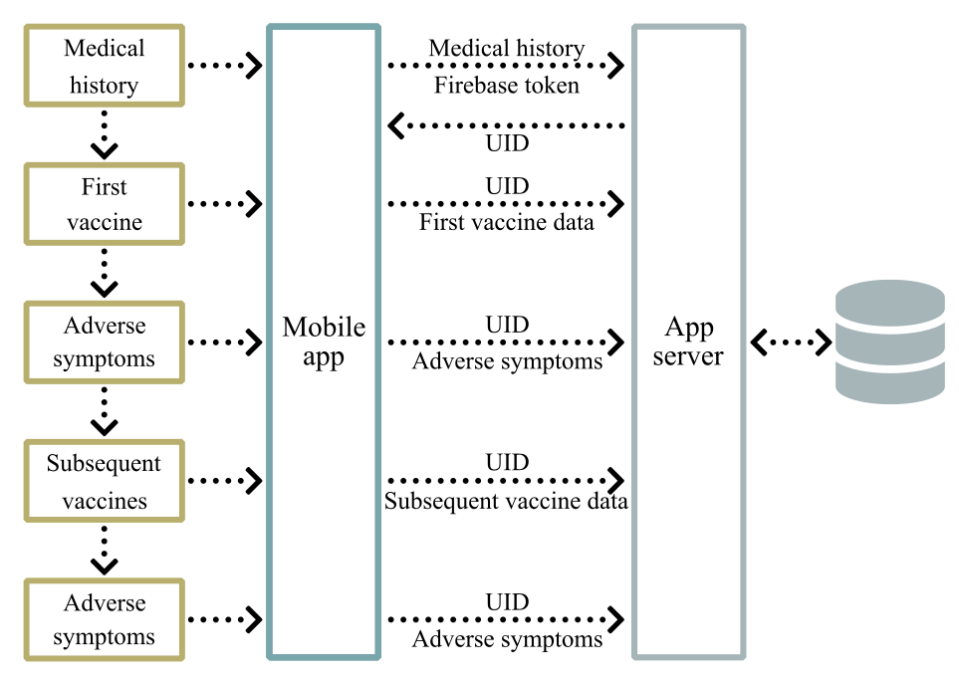
Our system’s data exchange. UID: user identifier.

The complete data exchange between the mobile app and the application server is described in Figure 2.

### Security by Design

The framework for recording the responses provided by the participants is a one-way client/server architecture. We decided to opt for this type of structure to ensure the safety of the respondents. This way, it was not necessary to store the answers to the questionnaires on the terminal used by the respondents, minimizing the possibility of illegitimate acquisition of information in the event of loss, theft, or fraudulent use of the device.

Furthermore, to ensure the anonymity of the respondents, while preserving the possibility of analyzing the time series relating to the same individual, we decided to generate a unique user identifier (UUID) using an FCM [62] token so that the UUID could not be used to trace the user of the mobile device (the FCM token identifies a single app on a single device and is not associated with any sensitive user data).

### Mobile App

The mobile app was developed for both Android and iOS devices using the FCM software development kit (SDK). We specifically used Android Studio (Java language) and XCode (Swift language) for, respectively, the Android and iOS versions. Our app sends data over the web to an application server using an application programming interface (API) gateway. The data are sent using the https protocol and are validated by the application server.

**Figure 3:**
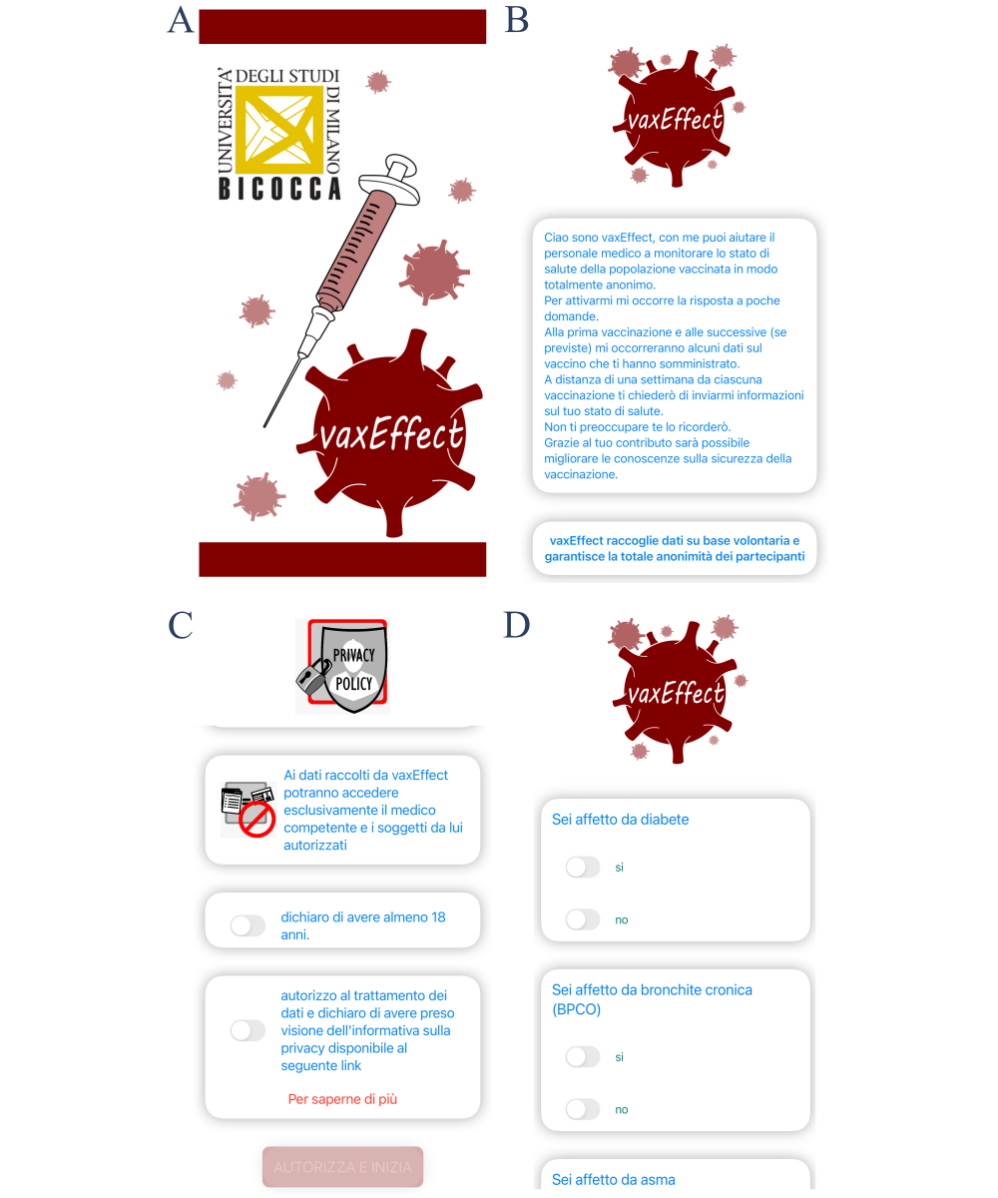
Mobile app first startup. (A) Splash screen. (B) Mobile app description. In this panel, the app is presented, specifying that the user will be asked for vaccination data after the first and, if any, every subsequent vaccination. Seven days after each vaccination, the user will be then asked to submit ADRs. Alerts will be sent to remind the user about the data submission after each moment. In addition, this panel specifies that data are collected on a voluntary basis, ensuring total anonymity of the submitted data. (C) Privacy. In this panel, it is specified that the architecture underlying vaxEffect@UniMiB ensures the privacy of the submitted data, which can be viewed by only the responsible physician and authorized subjects. Moreover, the user needs to confirm to be of legal age, to authorize the processing of the data that will be submitted, and to have read the provided privacy statements. (D) Medical history. At this stage, the user has to complete the medical history questionnaire by inserting demographic data and indicating whether 1 or more chronic diseases among those specified in the “Data collected by the app” section have been ever diagnosed. In this panel, an example of the data that the user needs to submit at this stage is provided by asking whether the user suffers from diabetes, bronchitis, or asthma. ADR: adverse drug reaction.

On initial startup of our mobile app, a splash screen is displayed (Figure 3A), and at the same time, the FCM SDK generates a registration token for the client app instance. This token uniquely identifies an app installation and cannot be linked in any way to the device owner, and it is used to receive a push notification from the application server. After the splash screen, a short description of the mobile app (Figure 3B), the privacy agreement (Figure 3C), and a medical history questionnaire (Figure 3D) are displayed. Medical history data and the registration token are sent to the application server. The application server stores the data received in the database and returns a UUID, which is encrypted and saved in the local mobile app file system.

**Figure 4:**
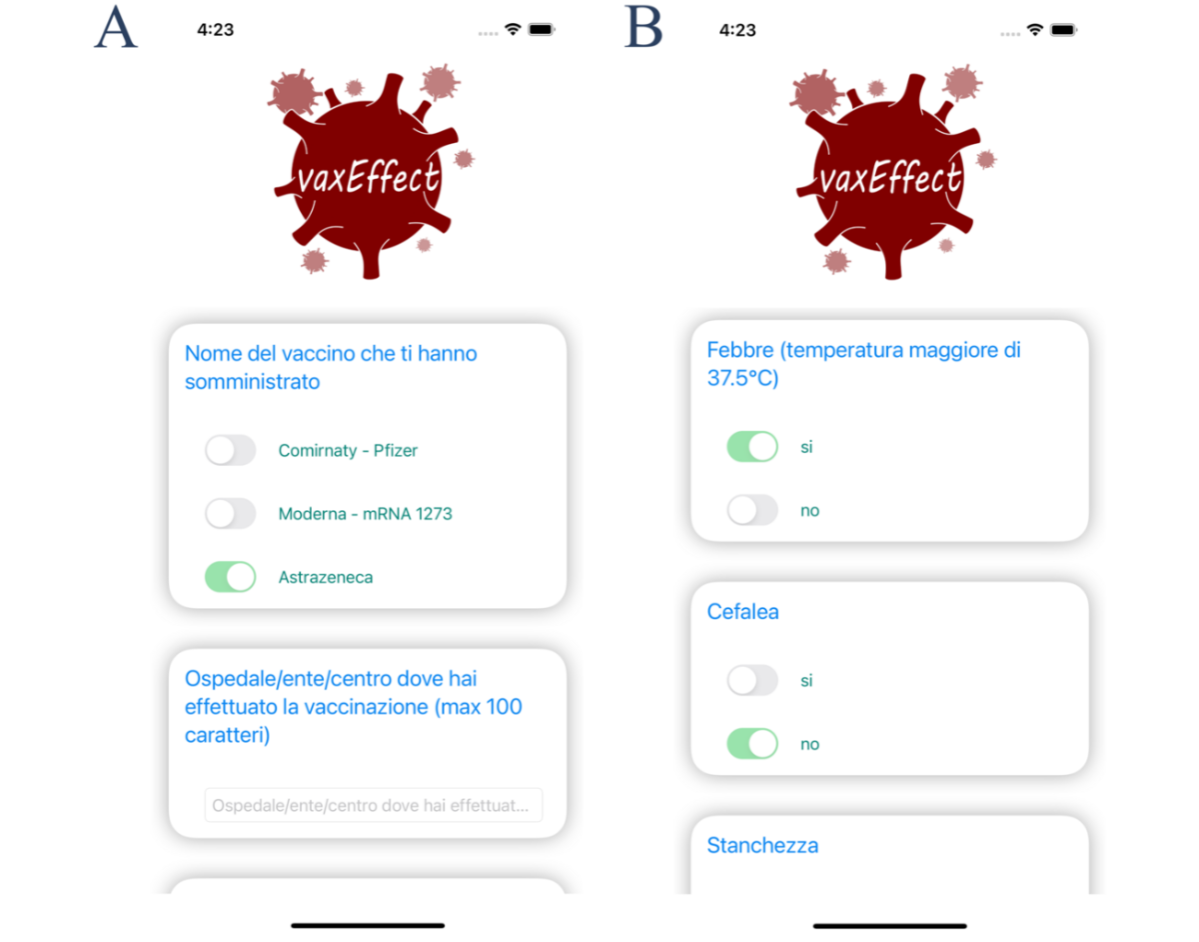
Mobile app at subsequent startup. (A) Vaccine data. After each vaccination, the app asks the user to submit vaccination data. In this panel, an example of the data that the user needs to submit at this stage is provided by asking the name of the administered vaccine (choosing among Comirnaty, moderna-mRNA1273, or Vaxzevria) and the hospital or institution where the vaccine has been administered. (B) ADRs. Seven days after each vaccination, the user will be asked to submit specific ADRs among those reported in the “Data collected by the app” section. In this panel, an example of the data that the user needs to submit at this stage is provided by asking whether fever, headache, or tiredness has occurred after vaccination. ADR: adverse drug reaction.

On subsequent startup or after completing the medical history questionnaire, the patient is asked for vaccination data (Figure 4A) and, 7 days later, for ADRs (Figure 4B).

### Application Server

The application server was developed in Python. Its main purpose is to collect data from the mobile app and store them in the database. The application server is also responsible for generating the UUID, to pair it with the FCM token, and to send push notification requests to the FCM server. Indeed, according to the data provided by the patient, after 7 days from the date of vaccination, the application server sends a push notification request to the FCM server in order to inform the patient that the ADR data need to be collected. The notification messages delivered to the mobile app from the FCM server open when the patient taps on the notification.

### Data Collected by the App

In addition to demographic data, the medical history questionnaire that respondents had to complete required them to indicate whether they had been diagnosed with one or more chronic diseases among those suggested by the app: dyslipidemia, hypertension, autoimmune diseases, asthma, cardiovascular diseases, tumor, liver diseases, diabetes, bronchitis, or kidney disease.

In addition, vaxEffect@UniMiB asks users to register the occurrence of specific ADRs among those reported as a COVID-19 vaccine side effect by the Centers for Disease Control and Prevention (CDC) and WHO: fever, headache, injection site pain, tiredness, muscular pain, swollen lymph nodes, joint pain, paresthesia, dizziness, sleepiness, nausea, and abdominal pain. At the same stage, vaxEffect@UniMiB collects information about the vaccine administrated to the subject, such as its kind: Vaxzevria (AstraZeneca), Comirnaty (Pfizer/BioNTech), moderna-mRNA1273 (Moderna), or Janssen (Johnson & Johnson).

### Data Analysis

To avoid cases of intentional or unintentional false reports by users, we checked the consistency of the submitted data at different time points, discarding nonconsistent data. Due the free access to the app and the data themselves, it was not possible to automatically detect fraudulent data reporting, but during a manual inspection, no fraudulent report was found.

For each user, we labeled the registered data, defining whether they belonged to the first, second, or third vaccination dose. By keeping separate the 3 sets of records, we analyzed in each the number of ADRs registered by each user by separating into 2 groups subjects who reported more than 3 postvaccination symptoms and those who reported fewer than 3 symptoms. These 2 groups of subjects were compared in terms of sex and age by using the chi-square test [63].

Through radar plots, we analyzed the kinds of ADRs reported by each user after every vaccine dose by keeping subjects separated in terms of gender and age.

Using a Sankey diagram [64], we followed the ADRs reported by users who submitted their data after all 3 vaccine doses, classifying who registered 0, 1, 2, 3, or more than 3 postvaccination symptoms.

## Results

### Characterization of vaxEffect@UniMiB Users

The total number of people vaccinated during the first wave of vaccine administration (March 2021) conducted at the Bicocca hub was 3712 (a third of the entire personnel of UniMiB and PoliMi). This partial coverage of the personnel can be ascribed to the fact that health care workers received the vaccine before the launching of the app, with the effect that not everyone may have necessarily participated in the survey presented in this work.

During this wave, vaxEffect@UniMiB received a total of 2733 medical history questionnaires from individuals between the ages of 19 and 80 years. In particular, as shown in the Medical History column of Tables 1-3, a greater involvement of UniMiB users compared to PoliMi users resulted in more usage of the app (n=1676, 61.3%, vs n=1057, 38.7%) at UniMiB due to how vaccinations at the UniMiB hub were organized. Indeed, all the UniMiB employees were allowed to get vaccinated at the hub. On the contrary, only part of PoliMi employees were allowed to get vaccinated at this hub.

**Figure 5:**
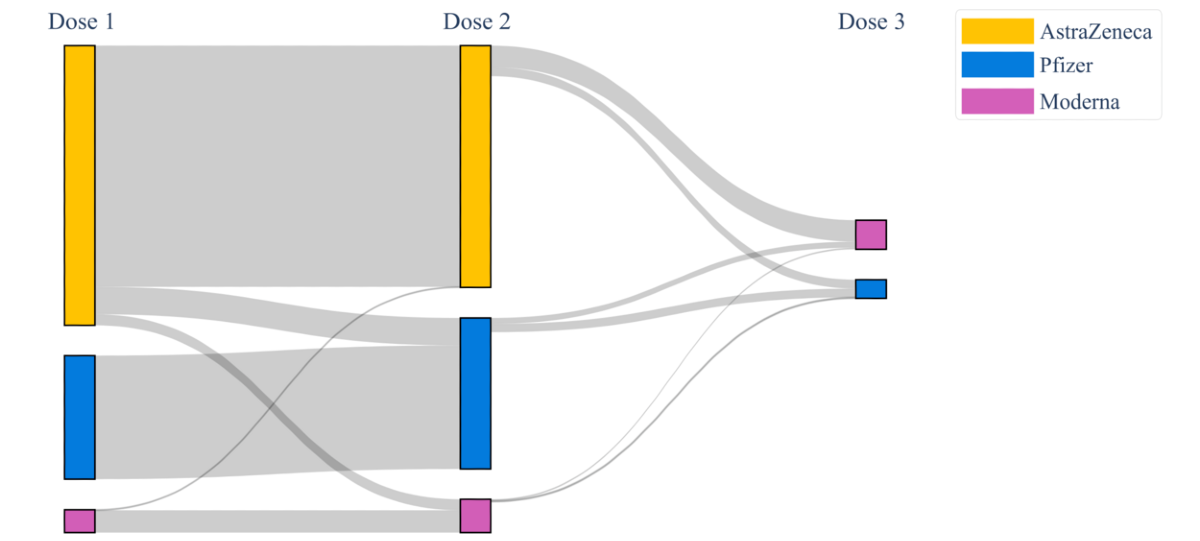
Kinds of vaccines administered to the same subject through the 3 vaccination campaigns.

vaxEffect@UniMiB allows characterizing the vaccinated population by the kind of administered vaccine. It is possible to observe that the administered vaccines were Vaxzevria (n=2711, 62.8%), Comirnaty (n=1253, 29.0%), moderna-mRNA1273 (n=349, 8.1%), and Janssen (n=5, 0.1%). The collected data made it possible to follow the proportion of each vaccine kind through the 3 doses (Figure 5).

**Table 1:** Characterization of vaxEffect@UniMiB users for the institution stratification.^a^

Institution	Medical history (N=2733), n (%)	Dose 1			Dose 2			Dose 3	
		Admin.^b^ (N=2360), n (%)	ADR^c^ (N=2226), n (%)	Admin. (N=1779), n (%)		ADR (N=1610), n (%)	Admin. (N=179), n (%)		ADR (N=169), n (%)
UniMiB^d^	1676 (61.3)	1383 (58.6)	1332 (59.8)	1252 (70.4)		1138 (70.7)	116 (64.8)		111 (65.7)
PoliMi^e^	1057 (38.7)	977 (41.1)	894 (40.2)	527 (29.6)		472 (29.3)	63 (35.2)		58 (34.3)

^a^Each cell reports the absolute and the relative frequency computed for the sample size N reported in the column headings.

^b^Admin*.*: individuals who submitted their vaccination data.

^c^ADR: adverse drug reaction (individuals who registered their ADRs 7 days postvaccination).

^d^UniMiB: University of Milano-Bicocca.

^e^PoliMi: Politecnico of Milan.

**Table 2:** Characterization of vaxEffect@UniMiB users for the gender stratification.^a^

Gender	Medical history (N=2733), n (%)	Dose 1			Dose 2			Dose 3	
		Admin.^b^ (N=2360), n (%)	ADR^c^ (N=2226), n (%)	Admin. (N=1779), n (%)		ADR (N=1610), n (%)	Admin. (N=179), n (%)		ADR (N=169), n (%)
Male	1224 (44.8)	1075 (45.6)	998 (44.8)	713 (40.1)		645 (40.1)	68 (38.0)		65 (38.5)
Female	1509 (55.2)	1285 (54.4)	1228 (55.2)	1066 (59.9)		965 (59.9)	111 (62.0)		104 (61.5)

^a^Each cell reports the absolute and the relative frequency computed for the sample size N reported in the column headings.

^b^Admin*.*: individuals who submitted their vaccination data.

^c^ADR: adverse drug reaction (individuals who registered their ADRs 7 days postvaccination).

**Table 3:** Characterization of vaxEffect@UniMiB users for the age stratification.^a^

Age (years)	Medical history (N=2733), n (%)	Dose 1			Dose 2			Dose 3	
		Admin.^b^ (N=2360), n (%)	ADR^c^ (N=2226), n (%)	Admin. (N=1779), n (%)		ADR (N=1610), n (%)	Admin. (N=179), n (%)		ADR (N=169), n (%)
<30	1301 (47.6)	1024 (43.4)	962 (43.2)	955 (53.7)		861 (53.5)	64 (35.8)		59 (34.9)
30-55	1109 (40.6)	1043 (44.2)	983 (44.2)	634 (35.6)		575 (35.7)	80 (44.7)		76 (45.0)
>55	323 (11.8)	293 (12.4)	281 (12.6)	190 (10.7)		174 (10.8)	35 (19.5)		34 (20.1)

^a^Each cell reports the absolute and the relative frequency computed for the sample size N reported in the column headings.

^b^Admin*.*: individuals who submitted their vaccination data.

^c^ADR: adverse drug reaction (individuals who registered their ADRs 7 days postvaccination).

Stratification of subjects by gender revealed a slightly greater proportion of female (n=1509, 55.2%) compared to male (n=1224, 44.8%) subjects. When stratified by age, younger (<30 years) and middle-aged (30-55 years) subjects represented the most active users, together constituting 88.2% (n=2410) of the total participants. On the contrary, older users (>55 years) represented just 11.8% (n=323) of the total set of survey respondents. Age and sex distributions of users employed in UniMiB were in line with those in PoliMi.

Once the medical history questionnaire was completed, 2360 (86.3%) and 1779 (65.1%) of all survey participants continued to use vaxEffect@UniMiB, reporting their vaccination data of, respectively, the first and the second dose campaign. Subsequently, 2226 (94.3%) and 1610 (90.5%) users who submitted their vaccination data after, respectively, the first and the second doses also reported their ADRs to the vaccination.

In response to the first vaccine dose, we collected information about vaccination and ADRs for 2360 and 2226 subjects (see the Dose 1 column in Tables 1-3), which represented, respectively, 63.6% and 59.9% of the 3712 individuals who were vaccinated during the first dose campaign. After the second vaccine dose, we collected information about vaccination and ADRs for 1779 and 1610 subjects (see the Dose 2 column in Tables 1-3), which represented, respectively, 47.8% and 43.3% of the 3718 individuals who were vaccinated during the second dose campaign.

The Dose 3 column in Tables 1-3 reports the same information relative to the administration of the third vaccine dose. Users who went further in reporting their data were considerably lower in number compared to the first two dose campaigns, since 179 users spontaneously continued to use vaxEffect@UniMiB to report their vaccination data and 169 of them also submitted their postvaccination ADRs. In this vaccination campaign, differently from the previous 2 ones, UniMiB was no longer the vaccination hub.

**Figure 6:**
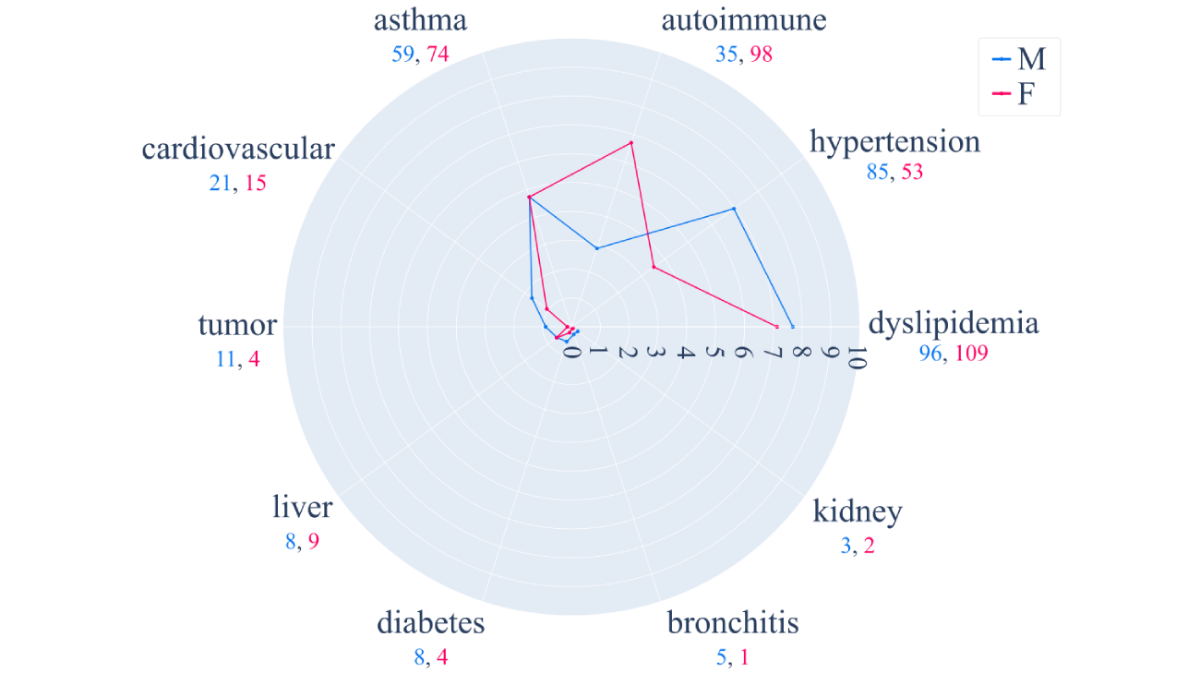
Prevalence expressed in percentage of chronic diseases among male (M) and female (F) users of vaxEffect@UniMiB. The blue and pink lines depict the percentage prevalence of each single chronic disease between, respectively, male and female subjects. Below each label, the corresponding absolute count in male and female users is reported colored according to the color scale on the right.

Stratifying the subjects who participated in the survey by gender (Figure 6) the prevalence of chronic diseases was overall fairly low since a maximum value of 0.078 was registered. Exploring the inserted data, the prevalence of the registered chronic diseases was higher in male than in female users, except for asthma, liver disorders, and autoimmune diseases. Moreover, focusing on cases where the gap between males and females was wider, we found that male subjects suffer more from hypertension, whereas females are more prone to autoimmune diseases.

### Characterization of ADRs After Each Vaccination Dose

**Figure 7:**
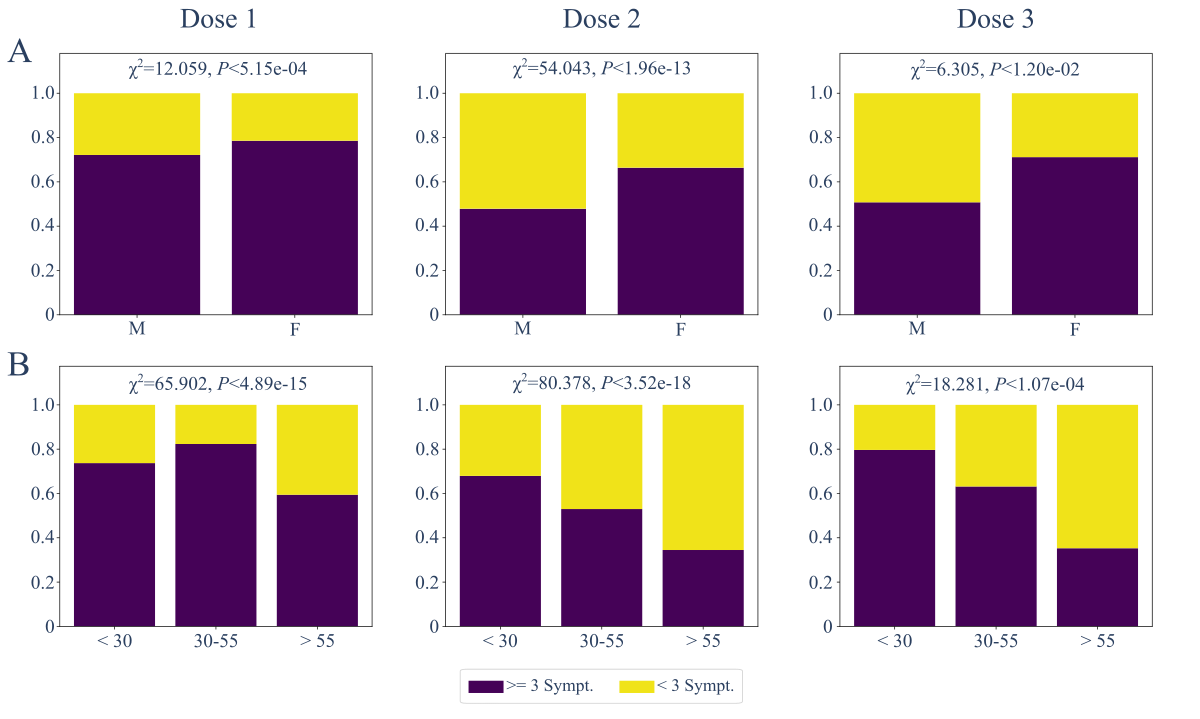
Number of ADRs registered by users after each vaccination dose. Subjects separately stratified by gender (A) and age (B) were grouped according to the number of postvaccination symptoms (>=3 Sympt. and <3 Sympt.). On the top of each plot, the result of the chi-square test and the corresponding *P* value are reported. ADR: adverse drug reaction.

As shown in Figure 7, independently of the type of vaccine and the dose administered, the response of subjects in terms of the number of reported ADRs to vaccination depended on gender and age. Indeed, females were more prone to a worse response to the vaccine, given the constant higher proportion of females compared to males who registered more than 3 symptoms/week postvaccination. In terms of age, the prevalence of ADRs to vaccination turned out to be worse in younger and middle-aged respondents compared to older users.

**Figure 8:**
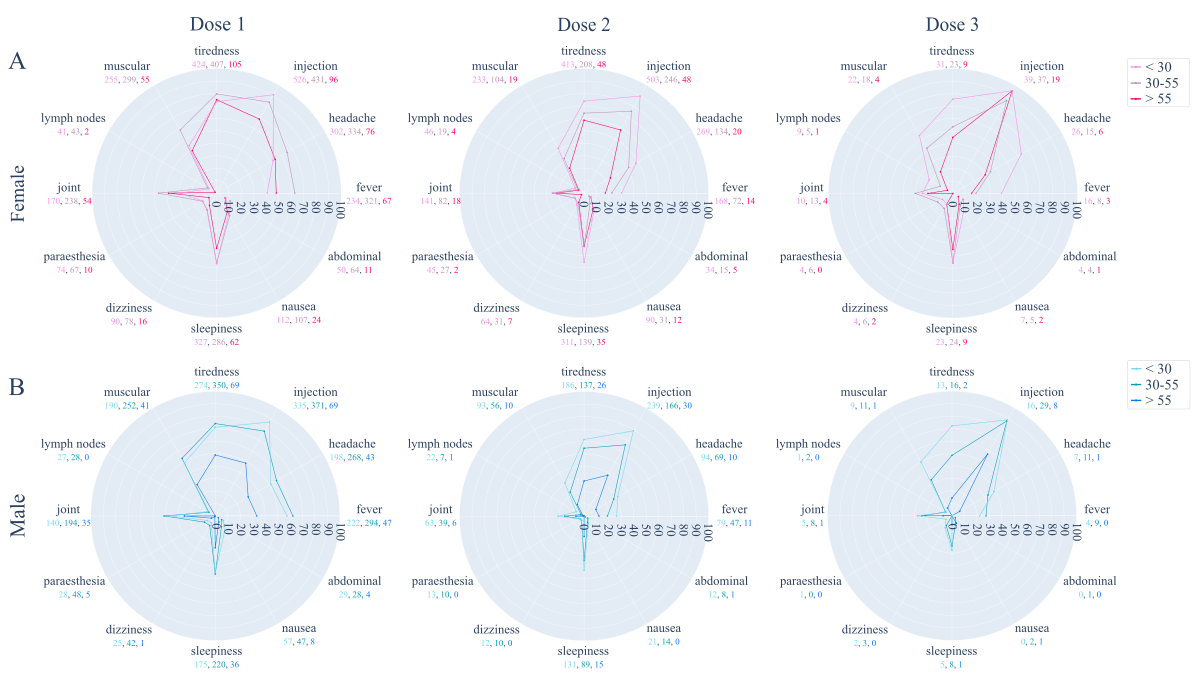
Kinds of ADRs registered by users after each vaccination dose. (A) Light-, middle-, and dark-pink lines denote the percentage prevalence of ADRs reported, respectively, by younger (labeled as <30 years), middle-aged (labeled as 30-55 years), and older (labeled as >55 years) female users. (B) Light-, middle-, and dark-blue lines denote the percentage prevalence of ADRs reported, respectively, by younger (labeled as <30 years), middle-aged (labeled as 30-55 years), and older (labeled as >55 years) male users. Below each label, the corresponding absolute count in younger, middle-aged, and older users is reported for females and males, colored according to the color scale on the right. ADR: adverse drug reaction.

Moving on to a more in-depth analysis of the kinds of symptoms reported after each vaccination dose, the most common vaccine ADRs were fever, headache, injection site pain, tiredness, muscular pain, joint pain, and sleepiness (Figure 8).

The types of ADRs to vaccine administration were dependent on both the gender and age of the participants. When analyzing the response of males and females separately, a progressive reduction in ADR prevalence through the 3 vaccine doses emerged for all the 3 age groups. However, we noted an exacerbation after the third vaccination dose for some of them. Specifically, among female subjects, more cases of fever, headache, injection site pain, and muscular pain were reported by young users; more cases of injection site pain, muscular pain, joint pain, and sleepiness by middle-aged users; and more cases of headache and injection site pain by older users. Instead, among male subjects, more cases of headache, injection site pain, tiredness, muscular pain, and joint pain were reported by young users; more cases of fever, headache, injection site pain, muscular pain, and joint pain by middle-aged users; and more cases of injection site pain by older users. Comparing males and females within the same vaccination dose, the same behavior as seen in Figure 7 emerged since females complained about more ADRs after each vaccine administration compared to male participants.

### Longitudinal Analysis of ADRs Through the 3 Vaccine Doses

The innovative feature that distinguishes vaxEffect@UniMiB from the existing mobile apps aimed at reporting ADRs is the ability to perform a longitudinal analysis to monitor the same subject over time in terms of the reported ADRs after each vaccine administration, while guaranteeing anonymity of the submitted data.

**Figure 9:**
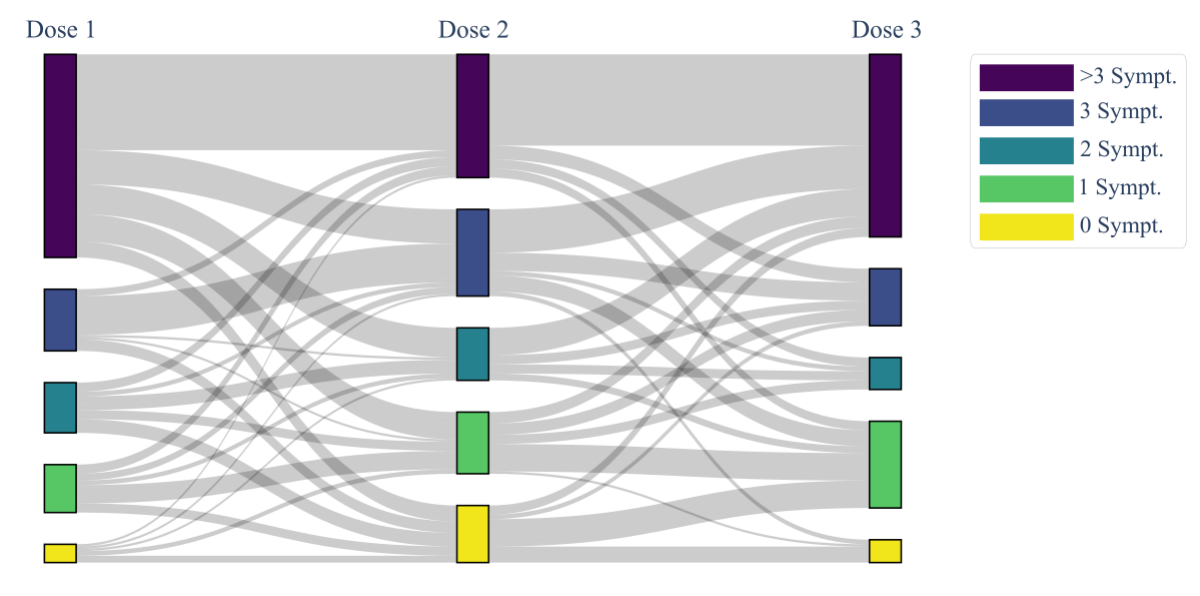
Longitudinal analysis of ADRs through the 3 vaccine doses. Each level identifies each of the 3 vaccine doses, respectively, labeled as Dose 1, Dose 2, and Dose 3. Each node identifies the 5 classes of symptom abundance, respectively, labeled as 0 Sympt., 1 Sympt., 2 Sympt., 3 Sympt., and >3 Sympt. and colored according to the legend on the right. The width of the links is proportional to the amount of subjects who experienced a given number of ADRs between Dose 1 and Dose 2 and between Dose 2 and Dose 3. ADR: adverse drug reaction.

In the overall cohort of the survey presented in this work, 167 users regularly submitted their ADRs after each of the 3 doses, enabling us to detect how the response of individuals changes depending on the number of ADRs registered after each dose (Figure 9).

## Discussion

### Principal Results

We proposed a new mobile app called vaxEffect@UniMiB, able to perform anonymized longitudinal studies, that, combined with the health care workers’ sponsorships, fostered the spontaneous citizenship participation in COVID-19 vaccination ADR data collection. In this work, over 3712 subjects were vaccinated during the first dose at the Bicocca hub, and we received a total of 2733 (73.6%) reports.

It should be noted that health care workers, receiving the vaccine before the launching of the app, may not have necessarily participated in the survey presented in this work. Moreover, due to deputed authority emergency plan, the hub invited all UniMiB employees and only part of PoliMi ones, the others being distributed to different hubs. This aspect further affected the number of reports received over the total number of subjects invited to the vaccination campaign.

Thanks to vaxEffect@UniMiB, we collected vaccination and ADR data relative to the first vaccine dose for 2226 (60.0%) of the 3712 individuals vaccinated during the first dose campaign, to the second dose for 1610 (43.3%) of the 3718 individuals vaccinated during the second dose campaign, and, in a nonsponsored fashion, to the third dose for 169 (4.6%) individuals.

Although a direct comparison of our reporting rate against traditional ones is not possible, a reporting rate of 120 questionnaires submitted to the National Pharmacovigilance Network for any 100,000 doses administered was reported, according to the Annual Report of Aifa (Agenzia italiana del farmaco) related to the surveillance of COVID-19 vaccine safety during December 27, 2020-December 26, 2021.

Following a review of the currently available mobile apps for monitoring ADR reporting for medical devices, drugs, or vaccines, our app revealed to be one step ahead. Specifically, vaxEffect@UniMiB is the first successful attempt in tracking the same user over time and identifying putative temporal patterns in an anonymous fashion. With this ability, we were able to analyze in this study the trend that emerged through the 3 vaccine doses administered to the same user. The first vaccination resulted in the strongest response among individuals, given the high prevalence of subjects with 3 or more than 3 symptoms. As already evidenced by Haas et al [65], part of this high response to the vaccine could be due to a misattribution of commonly experienced and nonspecific symptoms, such as headache and tiredness, as specific reactions due to the vaccine administration, instead of a condition of anxiety and worry making people hyperalert to the occurrence of any possible adverse events. Among the 3 administered doses, the second one was the least symptomatic since a general decreased trend in the adverse events from the first to the second dose emerged. Finally, between the second and the third dose, there was a predominantly increased trend in the number of reported symptoms. This behavior might be due to a change in the vaccine type, since after the second dose, AstraZeneca has not been administered since. However, given the low number of subjects who regularly submitted their data after all the 3 vaccine doses, we are not in a position to determine whether this increased trend may for sure be due to the change in the vaccine type that occurred between the second and the third vaccination campaign.

A series of aspects contributed to a greater involvement from people in using this app to report their ADRs to vaccination.

First, the user-friendly and interactive graphics of vaxEffect@UniMiB allowed ease of use of the app, resulting in a positive response rate after both the first and the second vaccination campaign. Although the response after the third campaign was lower compared to the previous 2 ones, it could be still considered a positive result. Indeed, during the third vaccination campaign, any reminder notification was sent to users, denoting a completely spontaneous participation of subjects in that phase. This result indicates the effectiveness of vaxEffect@UniMiB as an ADR-reporting system, although the active contribution of an institutional sponsorship is necessary.

Another aspect that contributed to the increase in the usage of vaxEffect@UniMiB was its availability for both Android and iOS devices, which made it reachable to as many users as possible.

Even more importantly, the protection of the submitted information assured users that high standards of confidentiality were maintained during data reporting. The architecture underlying vaxEffect@UniMiB ensured the anonymity of all the survey participants and also the privacy of their data, minimizing the illegitimate acquisition of information in the case of loss, theft, or fraudulent use of the device. These actions allowed guaranteeing proper use of the outcomes of the research, while preserving the possibility of tracking the same subject over time.

### Limitations

Although these features allowed for a positive response from the public, we are also aware that following an even stronger sponsorship of the vaccination campaign, the 373 users who just compiled the medical questionnaire without then reporting any other data would continue using the app. Likewise, increased support in the dissemination of the app could encourage who partially submitted data for only 1 or 2 vaccine doses to complete the entire survey.

We intend to also clarify that since the study presented in this work was a pilot study conducted on a selected voluntary population, we cannot exclude that our statistical measures could be affected by volunteer bias. This bias can occur at all stages of the study, and differences between volunteers and the target population are not restricted to sociodemographic factors but can include attitudes toward the study and institutions involved. However, the likelihood of volunteer bias increases as the refusal rate to volunteer increases. We observed a high rate of participation and a low rate of attraction until the third vaccination wave (when the Bicocca hub was not more in place and the in-person stimulus ended), probably due to the endorsement of participants’ anonymity and confidentiality.

The impact of this bias is unknown, but the observed sample should be quite younger and have a higher level of education and income than the target population, showing a higher rate of participation. These characteristics have been associated with greater extension and severity, so we cannot exclude that we overestimated the prevalence of symptoms [66].

This work being a pilot study, we chose to restrict the analyses to the respondents explicitly imputable to the hub itself. Consequently, data entry was strictly controlled since only ADRs to the administered vaccination were asked to be submitted. In this way, we were sure that all the reported information was not false-true ADRs due to other medical conditions of the user. However, as a suggestion for the future, if vaxEffect@UniMiB will be used in a different and more open context, it will be necessary to integrate a preprocessing phase to tame the possible distortions induced by the noncontrolled environment in which respondents report information.

### Conclusion

The results of this study showed that it is possible to leverage active citizenship engagement to increase the ADR-reporting rate using an agile and pervasive tool, such as a mobile app.

The combination between the synergy of the physician who proposed the app and the flexibility of vaxEffect@UniMiB seems to be an excellent tool to ensure high response rates in situations in which a priori information is scarce and decisions need to be made in a short time.

In addition to the promising results so far obtained, this work highlighted some practices that need to be carried out to foster approach efficacity, such as conspicuous support in the dissemination of the app to encourage more subjects to report their ADRs and steady user engagement to sustain data submission through the entire vaccination treatment when only partial information is reported.

## Abbreviations

ADR: adverse drug reaction
FCM: Firebase Cloud Messaging
IVSS: integrated vaccine surveillance system
PoliMi: Politecnico of Milan
SDK: software development kit
UID: user identifier
UniMiB: University of Milano-Bicocca
UUID: unique user identifier
WEB-RADR: Web–Recognising Adverse Drug Reactions
WHO: World Health Organization
